# ULK1 and ULK2 are less redundant than previously thought: computational analysis uncovers distinct regulation and functions of these autophagy induction proteins

**DOI:** 10.1038/s41598-020-67780-2

**Published:** 2020-07-02

**Authors:** Amanda Demeter, Mari Carmen Romero-Mulero, Luca Csabai, Márton Ölbei, Padhmanand Sudhakar, Wilfried Haerty, Tamás Korcsmáros

**Affiliations:** 1 Earlham Institute, Norwich Research Park, Norwich, NR4 7UZ UK; 2 Quadram Institute Bioscience, Norwich Research Park, Norwich, NR4 7UQ UK; 3 Faculty of Biology, University of Seville, 41012 Seville, Spain; 4 Eötvös Loránd University, Budapest, 1117 Hungary

**Keywords:** Gene regulatory networks, Bioinformatics, Cellular signalling networks

## Abstract

Macroautophagy, the degradation of cytoplasmic content by lysosomal fusion, is an evolutionary conserved process promoting homeostasis and intracellular defence. Macroautophagy is initiated primarily by a complex containing ULK1 or ULK2 (two paralogs of the yeast Atg1 protein). To understand the differences between ULK1 and ULK2, we compared the human ULK1 and ULK2 proteins and their regulation. Despite the similarity in their enzymatic domain, we found that ULK1 and ULK2 have major differences in their autophagy-related interactors and their post-translational and transcriptional regulators. We identified 18 ULK1-specific and 7 ULK2-specific protein motifs serving as different interaction interfaces. We found that interactors of ULK1 and ULK2 all have different tissue-specific expressions partially contributing to diverse and ULK-specific interaction networks in various tissues. We identified three ULK1-specific and one ULK2-specific transcription factor binding sites, and eight sites shared by the regulatory region of both genes. Importantly, we found that both their post-translational and transcriptional regulators are involved in distinct biological processes—suggesting separate functions for ULK1 and ULK2. Unravelling differences between ULK1 and ULK2 could lead to a better understanding of how ULK-type specific dysregulation affects autophagy and other cellular processes that have been implicated in diseases such as inflammatory bowel disease and cancer.

## Introduction

Macroautophagy, hereafter referred to as autophagy, is a lysosome-dependent intracellular metabolic process. Autophagy is highly conserved in all eukaryotic cells, and contributes to maintaining energy homeostasis, generating nutrients following starvation and is crucial in promoting cell survival. Autophagy is present in cells at a basal level but it is also activated at a higher level as a response to stress^1,2^.

Upon autophagy induction, an isolation membrane (phagophore) sequesters a small portion of the cytoplasm, including soluble materials and organelles, to form the autophagosome. The autophagosome then fuses with the enzyme-containing lysosome to become an autolysosome and degrades the materials contained within it^1^. As observed first in yeast, induction of autophagy is governed by the induction complex formed by Atg proteins: Atg1-Atg13-Atg17-Atg31-Atg29. In mammalian cells, the complex is composed of Atg1 homologs (ULK1 or ULK2), the mammalian homolog of Atg13 (ATG13), RB1-inducible coiled-coil 1 (RB1CC1/FIP200) and ATG101^3–5^. Induction of autophagy is tightly regulated through the protein mechanistic target of rapamycin complex 1 (mTORC1): whilst complex-associated, mTORC1 phosphorylates ULK1/2 and ATG13, resulting in their inactivation. Nevertheless, when cells are treated with rapamycin or starved of nutrients, mTORC1 becomes separated from the induction complex, resulting in dephosphorylation of these proteins and consequent autophagy induction^6^. Interestingly, this type of autophagy induction is specific to higher eukaryotes and some of the protist, however, as we previously demonstrated, non-unikont protists (such as *Toxoplasma *spp., *Leishmania *spp., and *Plasmodium *spp.) lack the Atg1 complex, and induce their autophagy in different ways^7^.

**Figure 1:**
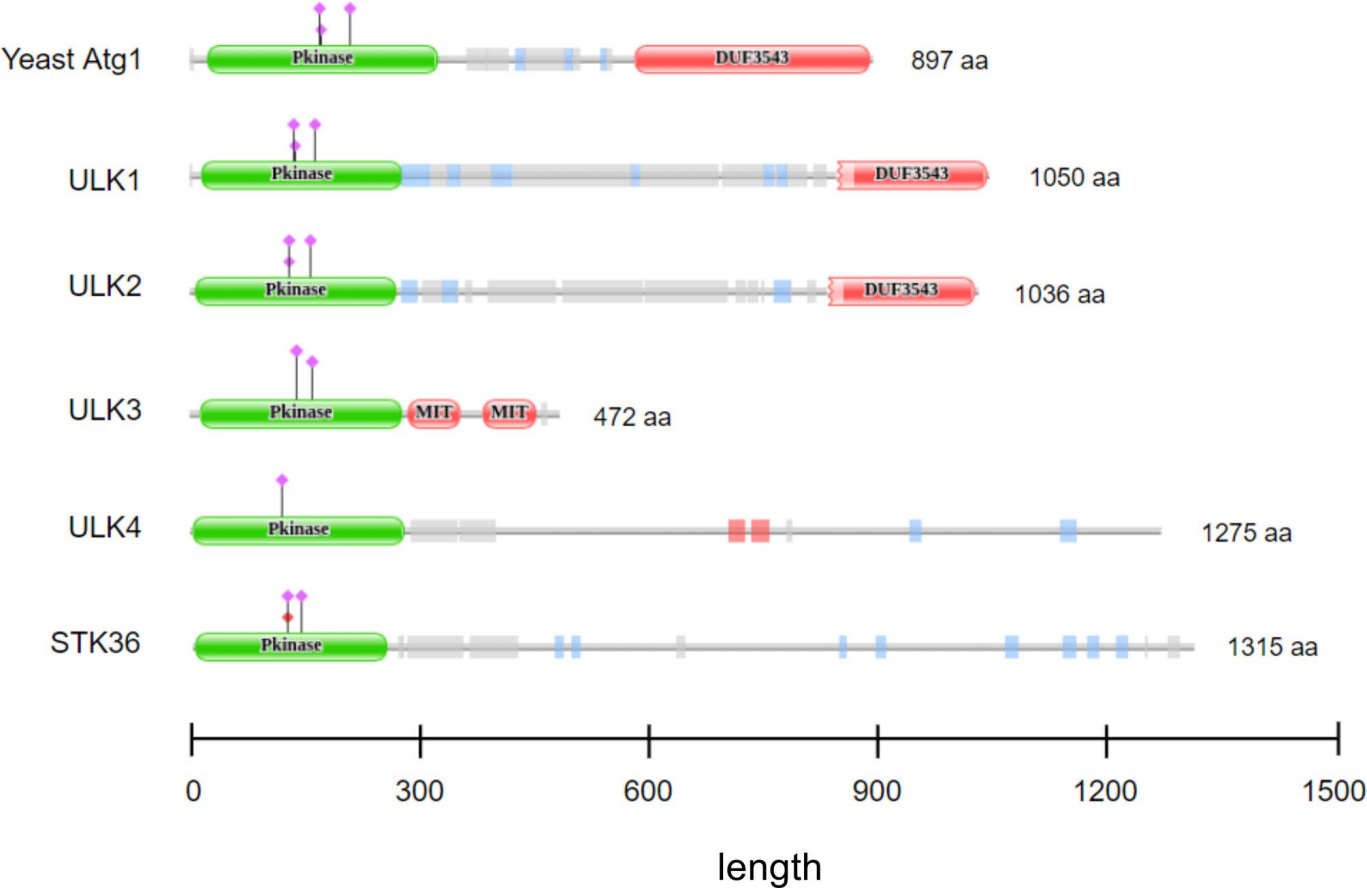
Domain structure of the yeast Atg1 protein and its human homologs. All Atg1 homologs have an N-terminal kinase domain, while only Atg1, ULK1 and ULK2 share the C-terminal DUF3543 domain. The domain structure of the proteins were downloaded from the Pfam website (https://pfam.xfam.org/)^47^.

The most studied component of the induction complex is the yeast *Atg1* and its homologs. There are five *Atg1* orthologs in the human genome: *ULK1, ULK2, ULK3, ULK4,* and *STK36*, all code proteins containing a kinase domain (Fig. 1.)^2,8^. Out of these genes, the protein product of *ULK1* and *ULK2* shows high similarity along the whole length of the protein (98% query cover but only 52% identity)^10^ and in their kinase domain (100% query cover with 78.71% identity)^2^. *ULK1* and *ULK2* genes code for serine/threonine protein kinases that consist of a conserved N-terminal kinase domain (catalytic domain), a central serine-proline rich region (PS), and a C-terminal interacting domain (CTD)^9^. ULK1 and ULK2 are best known to be involved in the induction of autophagy^10,11^, and are often mentioned interchangeably, however, there is growing evidence for functional differences, both in relation to autophagy and other functions. In different cell lines studies, loss of *ULK1* was found to result in interruption of starvation induced autophagy, and *ULK2* was thought to have a redundant function^12,13^. In cerebellar granule neurons, ULK1, but not ULK2, was also shown to be critical to induce the autophagic response^2^. Demonstrating the complexity of the relationship between ULK1 and ULK2, opposing evidence was found by Kundu et al*.*, who showed that deletion of *ULK1*, hence autophagy deficiency, resulted in delayed clearance of ribosomes and mitochondria in reticulocytes but in case of starvation-induced autophagy *ULK1* does not play an essential role^14^. Lee and Tournier also showed that the function of ULK2 is more likely to compensate for the loss of *ULK1* but in a cell-type specific manner^2^. Some speculations indicate that a dominance of either of the two ULK proteins is determined by differential tissue expression levels^15,16^. Regarding non autophagy-related differences, for instance, Seung-Hyun et al. found that while both ULK1 and ULK2 have shared functions in autophagy, they have opposing roles in lipid metabolism^10^. ULK1 and ULK2 were also shown to be part of different molecular complexes, possibly resulting in having independent functions or mode of regulation. It was specifically found that following amino acid starvation, in contrast to ULK1, binding of ULK2 to membranes was increased^17^. Finally, another difference between ULK1 and ULK2, described by Kundu et al. was, that during erythroid differentiation, *ULK1*, but not *ULK2* was found to be upregulated^14^. Showing the importance of both homologues, in mice studies, the knockout of both *ULK1* and *ULK2* showed neonatal lethality, which was seen also upon the loss of other autophagy genes such as *ATG5* and *ATG7*^14,15,18^.

The aim of these aforementioned experiments was in fact to compare ULK1 and ULK2 in specific areas, hence they included both proteins/genes in the experimental setup. However in other cases the experiment focused only one of the ULK homologs e.g. Refs^19–22^. In those cases, and when there is not enough information available about the experimental conditions, we cannot make an easy comparison between the function of ULK1 and ULK2. Altogether, even though experimentally validated interactions are more reliable than predictions, careful interpretation of the collected data is crucial.

Prompted by the evidence for the different roles of ULK1 and ULK2 in specific areas, we aimed to compare the two human paralogs on a systems-level. We aimed to do that based on experimentally validated, published interactions, tissue-specific RNA expression levels and structure-based predictions. We show that ULK1 and ULK2 are indeed different in their protein binding motifs and in their promoter sequences, harbouring binding sites for different transcription factors. ULK1 and ULK2 also have different experimentally validated autophagy-related and non-autophagy related interactors, and their post-translational and transcriptional regulators are relevant in distinct biological processes. With analysing the tissue-specific expression of genes encoding ULK1- and ULK2-specific interactors we found a diverse expression pattern, indicating a complex and distinctive interaction network for both ULK proteins. The balance between these two homologs and their specific processes can be crucial in diseases, especially when *ULK1*, *ULK2* or their interactors are differentially expressed.

## Results

### The duplication of the yeast *Atg1* happened at the base of the Chordates resulting in the most similar homologs, *ULK1* and *ULK2*

**Figure 2:**
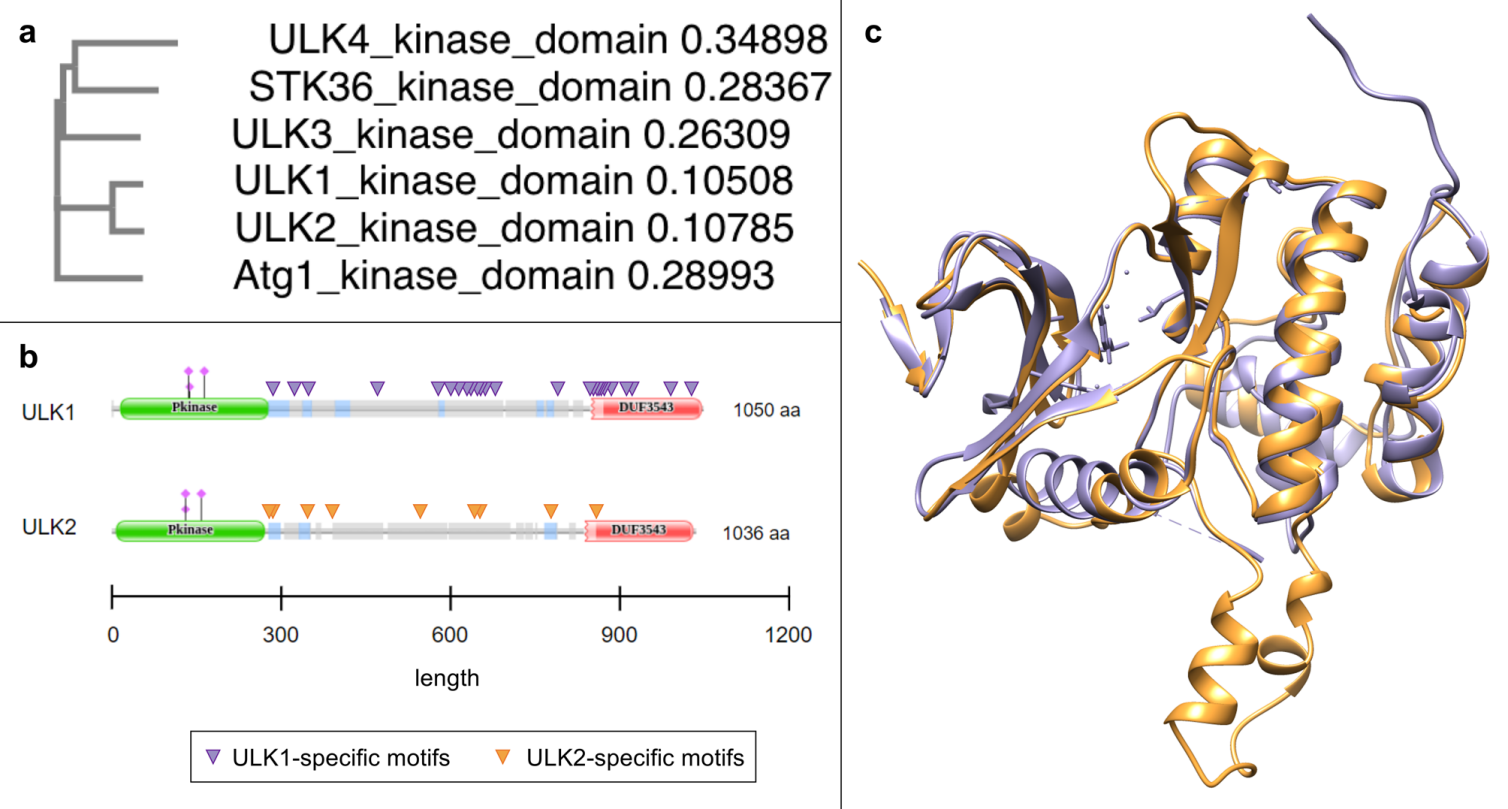
Alignment of kinase domains of the yeast Atg1 protein and its human homologs. (**a**) Based on multiple sequence alignment of the kinase domains ULK1 and ULK2 are the most similar to each other (similar results were obtained with multiple sequence alignment of the complete amino-acid sequences shown on Supplementary Figure S1). The protein sequences were downloaded from Uniprot (https://uniprot.org/)^50^ and the protein alignments were carried out with MUSCLE (https://ebi.ac.uk/Tools/msa/muscle/)^51^; (**b**) Unique motifs shown on ULK1 and ULK2 proteins; (**c**) Superimposition of 3D structures of ULK1 (purple) and ULK2 (orange) kinase domains shows aligning and non-aligning parts. 3D structure alignment was carried out with UCSF Chimera software^48^.

The multiple sequence alignment of the yeast Atg1 protein and its human orthologs shows that the human ULK1 and ULK2 are the most similar to each other (Fig. 2a). We obtained similar results with alignment of the kinase domain and alignment of the complete amino-acid sequences (Supplementary Figure S1). When comparing ULK1 and ULK2 one aspect was the accessible motifs along the proteins. In Fig. 2b, we visualized specific motifs that we identified as being specific to ULK1 or ULK2 (the function of the specific motifs is detailed in Tables 1 and 2). Despite the highest similarity among ULK homologs, superimposition of the 3D structures of ULK1 and ULK2 kinase domains shows both similar and non-aligning stretches (Fig. 2c).

**Table 1:** ULK1-specific motif details from the ELM resource (https://elm.eu.org/)^52^.

ULK1-specific motif name	Functional site class	Function
CLV_C14_Caspase3-7	Caspase cleavage motif	Caspase-3 and Caspase-7 cleavage in programmed cell death (apoptosis)
CLV_Separin_Metazoa	Separase cleavage motif	Sister chromatid separation in the metaphase-to-anaphase transition in cell division
DEG_APCC_DBOX_1	APCC-binding destruction motifs	Proteasome-dependent degradation of proteins in a cell cycle dependent manner by ubiquitination mediated by APC/C
DEG_COP1_1	COP1 E3 ligase binding degron motif	Proteasome-dependent degradation of proteins by ubiquitination mediated by COP1
DOC_ANK_TNKS_1	Tankyrase-binding motif	PARsylation of target proteins by Tankyrase regulating their ubiquitylation, stability and function
DOC_Cyclin_RxL_1	Cyclin docking motif	Protein phosphorylation by cyclin/Cdk complexes involved in different biological processes, such as stress response or cell division checkpoints
DOC_MAPK_FxFP_2	MAPK docking motifs	Increased binding affinity to specific interactors of the MAPK cascade, regulators of cellular signalling
LIG_Actin_WH2_2	Actin-binding motifs	Regulation of the actin filaments assembly influencing cellular functions such as the control of cell shape or cellular transport
LIG_BIR_III_2	IAP-binding motif (IBM)	Binding to Inhibitor of Apoptosis Proteins (IAPs) leading to apoptosis promotion
LIG_CtBP_PxDLS_1	CtBP ligand motif	Interaction and recruitment into nuclear complexes of proteins called CtBP (C-terminal binding protein), repressors of transcription
LIG_EVH1_1	EVH1 ligands	Binding to signal transduction class I EVH1 domains, influencing various signal transduction pathways, such as re-organization of the actin cytoskeleton
LIG_GBD_Chelix_1	GTPase-binding domain (GBD) ligand	Binding to the GTPase-binding domain (GBD) in WASP and N-WASP, thus preventing Arp2/3-dependent activation of actin polymerization
LIG_PDZ_Class_2	PDZ domain ligands	Recognition of short sequences at the carboxy terminus of target proteins, developing a variety of biological processes including cell signalling and synapse
LIG_Pex14_2	Pex14 ligand motif	Binding to a hydrophobic groove on Pex14, a key protein in peroxisomal import
LIG_SH3_1	SH3 ligand	Protein–protein interactions involved in several biological processes: signal transduction pathways, cytoskeleton organization, membrane traffic or organelle assembly
LIG_TYR_ITIM	Immunoreceptor tyrosine-based motif	Recruitment and activation of a protein tyrosine phosphatase that regulates the activation or inhibition of the intracellular response among immune cells (T, B and natural killer cells)
LIG_WW_3	WW domain ligands	Recognition of proteins that contain the motif PPR, being involved in many cellular processes such as ubiquitin-mediated protein degradation and mitotic regulation and several human diseases
MOD_NEK2_2	NEK2 phosphorylation site	NEK2 phosphorylation motif which can involve many cell cycle-related functions, including cell cycle progression or chromatin condensation

The list and annotated functions of 18 ULK1-specific motifs.

**Table 2:** ULK2-specific motif details from the ELM resource (https://elm.eu.org/)^52^.

ULK2-specific motif name	Functional site class	Function
DOC_MAPK_DCC_7	MAPK docking motifs	Interaction towards the ERK1/2 and p38 subfamilies of MAP kinases, regulators of cellular signalling
LIG_APCC_ABBA_1	APCC activator-binding ABBA motif	Regulation of APC/C activity in ubiquitination for proteasome-dependent degradation in a cycle-dependent manner
LIG_CaM_IQ_9	Helical calmodulin binding motifs	Primary receptor of intracellular Ca^2^ + capable of responding to a wide range of calcium concentration and translates the Ca^2^ + -signal into cellular processes
LIG_EF_ALG2_ABM_2	ALG-2 EF hand ligand	Binding to ALG-2 protein in a Ca2 + -dependent manner. ALG-2 has been implicated in ER-stress-induced apoptosis, cell cycle progression, the endosomal pathway, and cancer
LIG_SH2_GRB2like	Phosphotyrosine ligands bound by SH2 domains	Binding to specific motifs containing a phosphorylated tyrosine residue, propagating the signal downstream promoting protein–protein interaction and/or modifying enzymatic activities
LIG_TRAF6	TRAF6 binding site	Initiation of intracellular signalling caused by members of the tumor necrosis factor receptor (TNFR) superfamily, and direct interaction with intracellular regions of various TNF cytokine receptors
LIG_WW_1	WW domain ligands	Recognition of proteins that contain the motif PPXY, being involved in many cellular processes such as ubiquitin-mediated protein degradation and mitotic regulation and several human diseases

The list and annotated functions of 7 ULK2-specific motifs.

**Figure 3:**
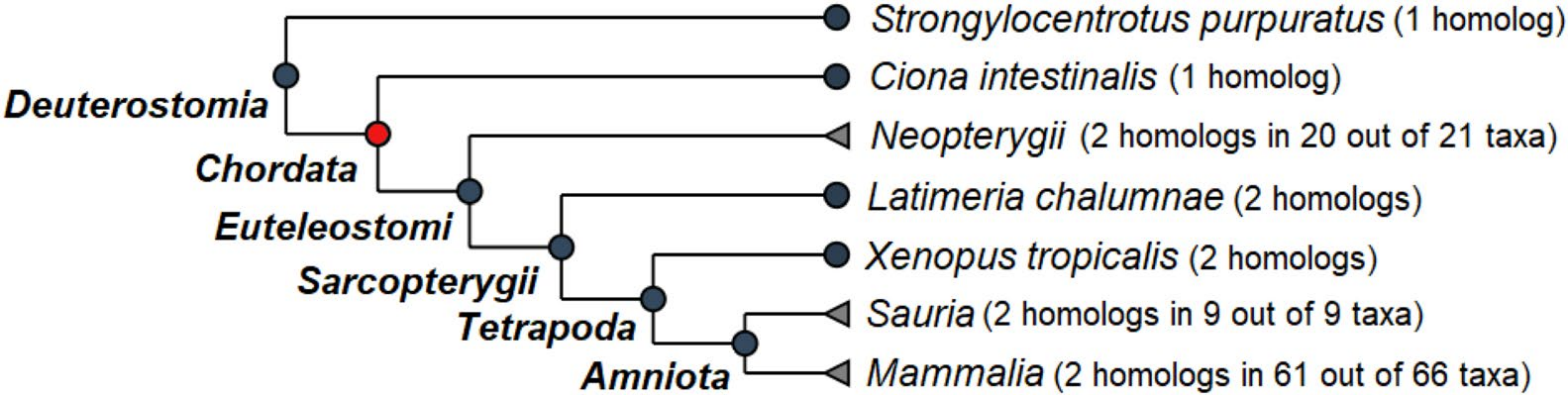
Phylogeny of the human *ULK1* gene. Duplication of the *ULK1* gene happened at the base of the Chordates, after the split of *Ciona intestinalis* from the Euteleostomes or “bony vertebrates”. The dendrogram was adapted from the OMA orthology browser (https://omabrowser.org/)^49^.

We searched for the orthologs of *ULK1* in other species to determine the first duplication event that gave rise to *ULK1* and *ULK2* (in humans). In Fig. 3, we visualize the duplication event which happened at the base of the Chordates, after the split of Urochordates (Tunicates, around 500 million years ago) from the Euteleostomes or “bony vertebrates”. As expected, in Deuterostomes and in *Ciona intestinalis* we can find only one ortholog of the human *ULK1* gene, but in the rest of the Chordates most taxa have two paralogs.

### Autophagy proteins interacting with ULK1 and ULK2 are involved in different types of selective autophagy

**Figure 4:**
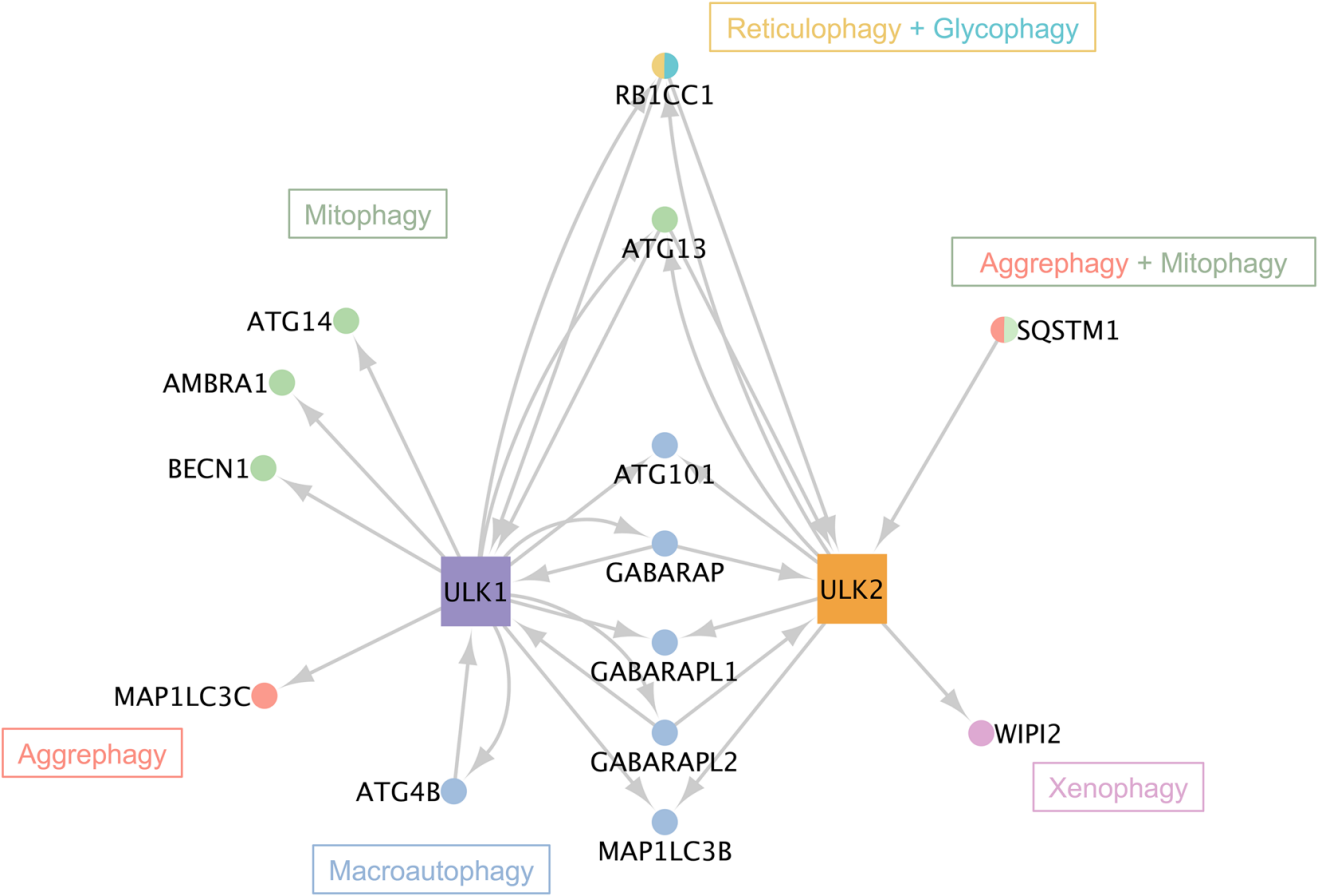
The autophagy protein interactors of ULK1 and ULK2 are involved in different types of selective autophagy. All of the interactors are associated with macroautophagy, but most of them affect one of two types of selective autophagy as well (mitophagy: green, aggrephagy: pink, reticulophagy: yellow, glycophagy: light blue, or xenophagy: purple).

By downloading data from the Autophagy Regulatory Network resource, we developed earlier^23^, and updating it with recent manually curated data, we collected direct interactions between ULK1, ULK2 and other autophagy proteins. Out of the 12 ULK1 first neighbours and the 9 ULK2 first neighbours, 5 and 2 are specific to ULK1 and ULK2, respectively (Fig. 4 and Supplementary Table S2). Based on the annotation of autophagy proteins in the Gene Ontology database and the work of Denny et al.^24^, we visualized specific and shared interactors of ULK1 and ULK2. All of these interactors are associated with macroautophagy, but there are differences in the type of selective autophagy for specific interactors of ULK1 and ULK2. For example, there is a shift in involvement in mitophagy (selective engulfment of mitochondria) towards ULK1-specific interactors, while the only protein that is connected to xenophagy (degradation of pathogens i.e. intracellular bacteria, viruses), WIPI2 is specific to ULK2. Reticulophagy (degradation of portions of the endoplasmic reticulum), glycophagy (degradation of glycogen) and aggrephagy (degradation of protein aggregates)^25^ are shared functions between ULK1 and ULK2.

### ULK1 and ULK2 have specific non-autophagy protein–protein interaction partners

**Figure 5:**
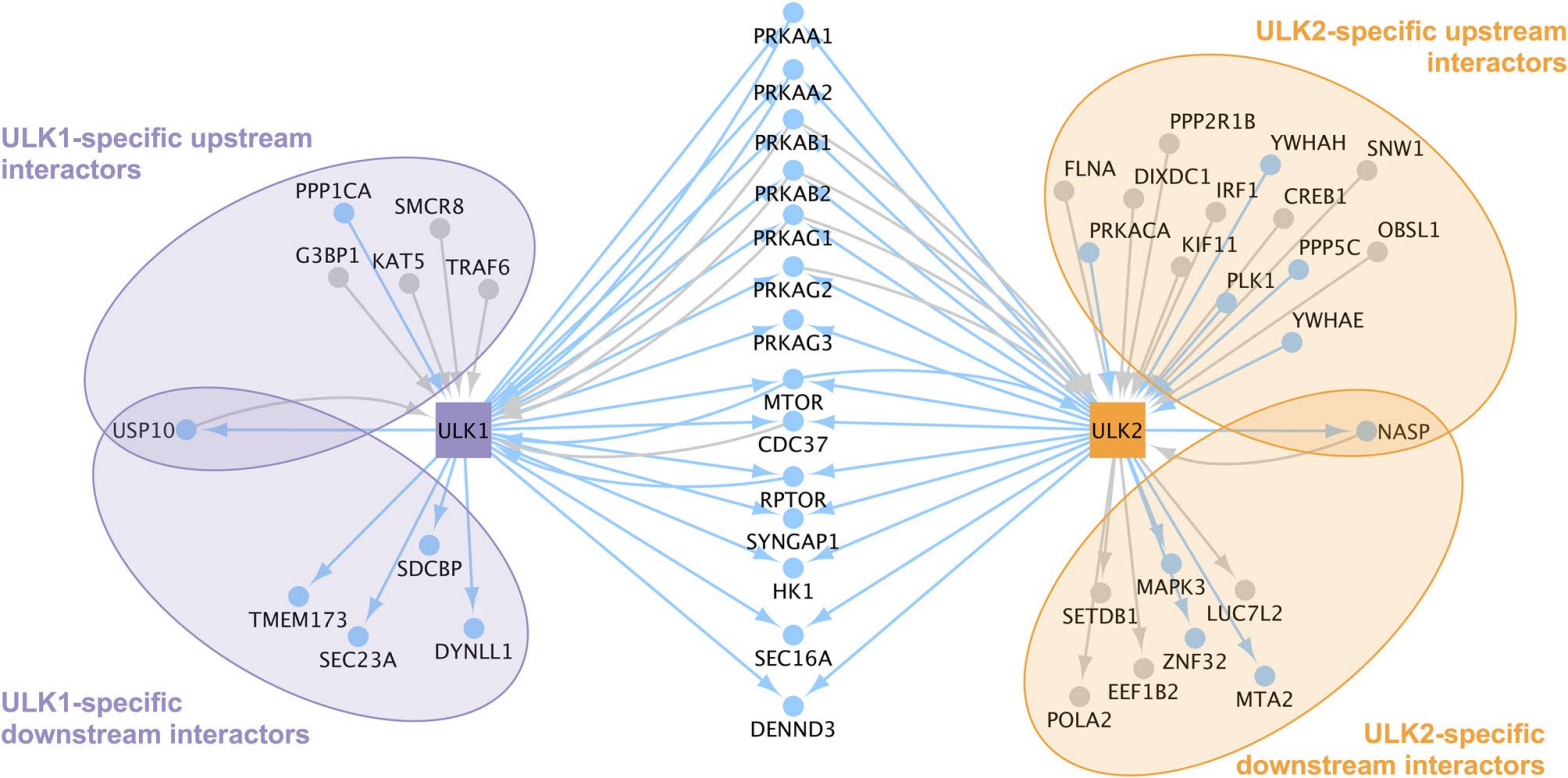
Experimentally validated protein–protein interactions of ULK1 and ULK2 collected from databases and from the literature by manual curation. Blue nodes and edges represent those experimentally identified interaction partners and interactions, respectively, that we found to be supported by a protein binding motif analysis (done with the ELM resource^52^). Grey nodes and edges show experimentally identified ULK-specific interaction partners and interactions, respectively for which the protein motif analysis did not provide potential binding interfaces.

We collected the experimentally verified protein interactors of ULK1 and ULK2 (termed as first neighbours) from databases and from the literature by manual curation. Out of the 25 ULK1 first neighbours and the 35 ULK2 first neighbours, 11 and 21 are specific to ULK1 and ULK2, respectively (Fig. 5 and Supplementary Table S2).

In Fig. 5, we visualized the experimentally validated directed interactions between the ULK proteins and their first neighbours. We grouped the neighbours based on being upstream or downstream from their respective ULK protein and based on being involved in single or bidirectional interaction. We also show that most of the first neighbours are predicted to bind to both ULK1 and ULK2, indicating a potential study bias in the literature data. The presence of the common downstream interactors in the prediction is possibly due to the domain similarity of the ULK homologs.

As both ULK1 and ULK2 have a kinase domain with 78.71% identity, we hypothesized that the explanation for having specific upstream interactors could be primarily due to exhibiting different protein motifs. Based on structural information, we found that beside sharing 37 motifs, ULK1 and ULK2 have 18 and 7 unique motifs, respectively (Tables 1, 2). Amongst the ULK1-specific motifs, we identified sites for caspase cleavage, sister chromatid separation and binding to inhibitor of apoptosis proteins, which could act in favour of promoting apoptosis. On the other hand, amongst the ULK2-specific motifs there are sites for deubiquitination and interaction with TNF cytokine receptors.

### ULK1 and ULK2 have tissue-specific interaction partners

We analysed the RNA expression of *ULK1, ULK2* and the genes encoding their specific protein interactors, and clustered them based on expression values across all assessed tissues (62 tissues were included). The interactors were not clustered based on their specificity to ULK1 or ULK2, and they were not necessarily expressed together with their respective ULK partner.

**Figure 6:**
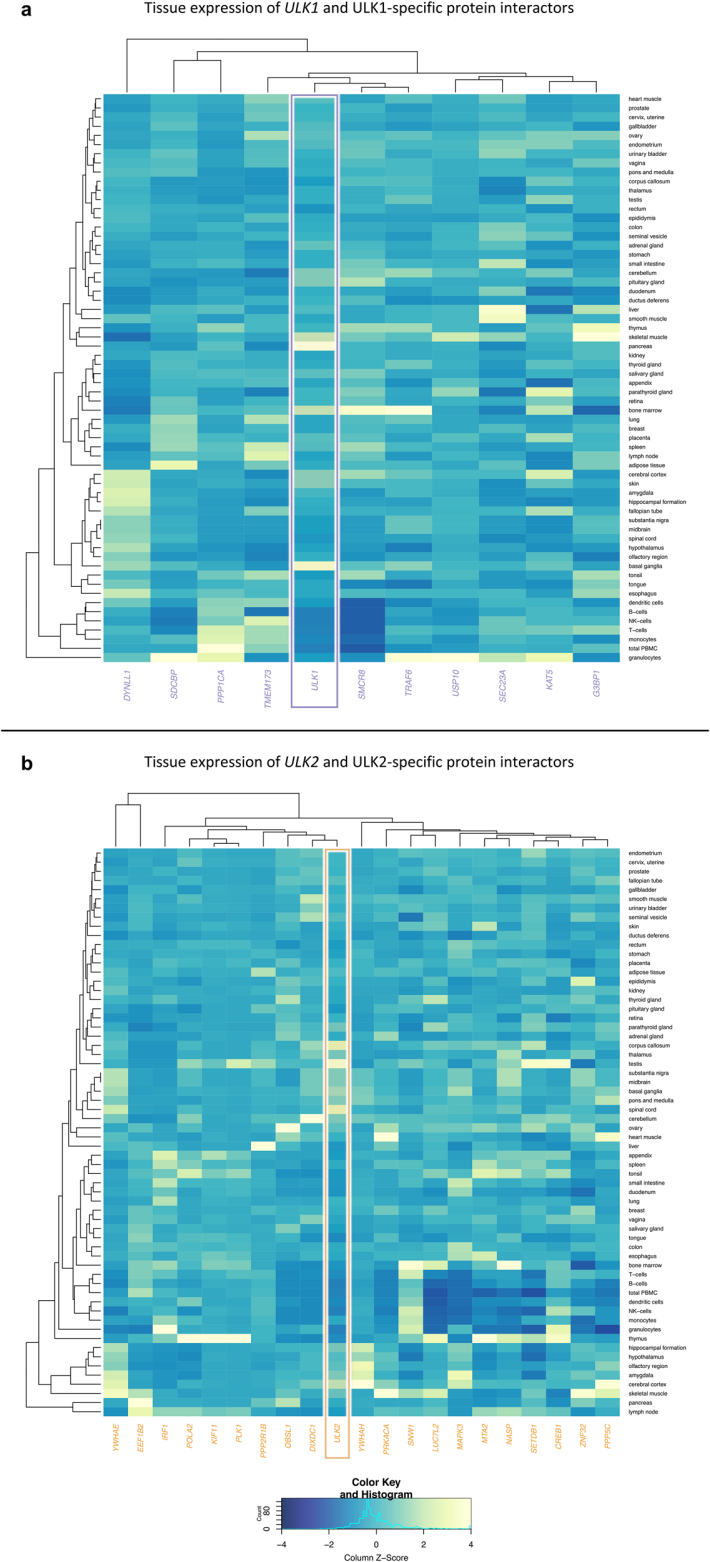
Interactors of ULK1 and ULK2 have tissue specific expression levels. (**a**) heatmap visualising expression of *ULK1* and genes encoding its specific interactors in 62 different tissues; (**b**) heatmap visualising expression of *ULK2* and genes encoding its specific interactors in 62 different tissues. The colour scale represents the relative expression of each interactor in different tissues (Column Z-score) (Purple: *ULK1* and its specific interactors; Orange: *ULK2* and its specific interactors).

*ULK1* and *ULK2* are generally similarly expressed in the analysed tissues, with only a few exceptions. *ULK1* is higher expressed in pancreas, skeletal muscle, basal ganglia and bone marrow, while *ULK2* is higher expressed in spinal cord, corpus callosum and testis (Supplementary Table S3). In Fig. 6, we visualized relative expression scores of *ULK1*, *ULK2* and their specific interactors. Some of the interactors (e.g. USP10, KIF11, PLK1) are similarly expressed (around column Z-score 0) in most of the tissues, the rest of them are more variable between tissues. Out of the ULK1- and ULK2-specific interactors only a few (SMCR8 and TRAF6; OBSL1 and DIXDC1) are clustered together with their respective *ULK* partners. Based on Fig. 6, even though there is no clear segregation between ULK1- and ULK2-specific interactors, both ULKs seems to have tissue-specific interaction partners.

### The non-autophagy first neighbour interactors of ULK1 and ULK2 are involved in different biological processes

**Figure 7:**
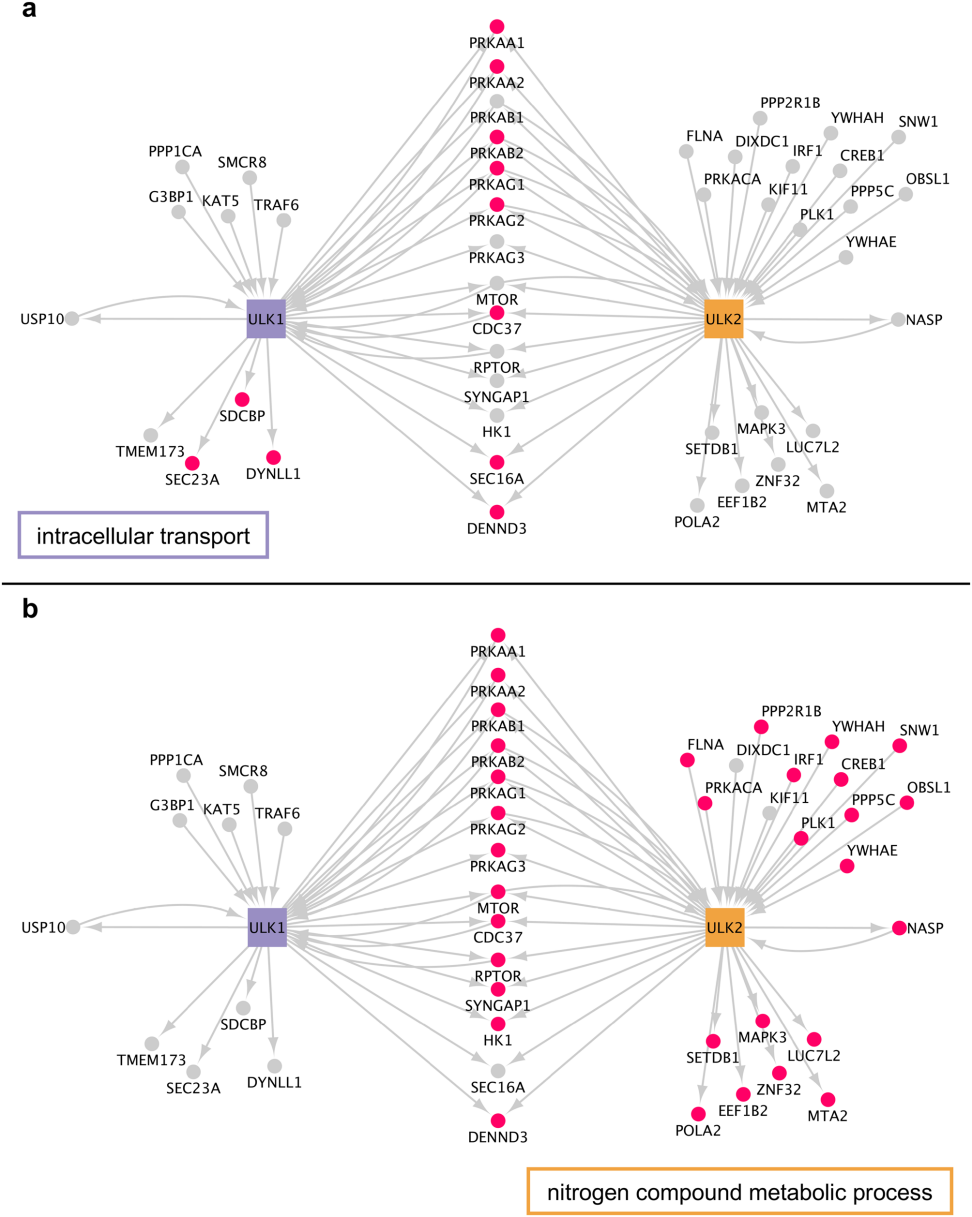
The protein–protein interaction partners of ULK1 and ULK2 and two biological processes predominantly being associated to ULK-specific protein interactors. The biological processes are labelled on each network, interactors involved in the respective biological process are highlighted with pink nodes. (**a**) Intracellular transport; (**b**) nitrogen compound metabolic process.

We analysed the shared biological processes within experimentally verified protein interactors of ULK1 and ULK2, respectively. Based on a Gene Ontology Term Finder analysis^26^, we found that beside the common biological functions, the interactors also seem to be responsible for specific processes. Based on our analysis, interactors of ULK1 share the function of intracellular transport (Bonferroni corrected *P* value for the hypergeometric distribution < 0.0001) with SEC23A, SDCBP and DYNLL1 being specifically interacting with ULK1. Meanwhile, ULK2 has interactors relevant in nitrogen compound metabolic processes (corrected *P* value < 0.0001) including 19 out of the 21 ULK2-specific interactors (Fig. 7).

### *ULK1* and *ULK2* have specific transcriptional regulators, which are also involved in different biological processes

**Figure 8:**
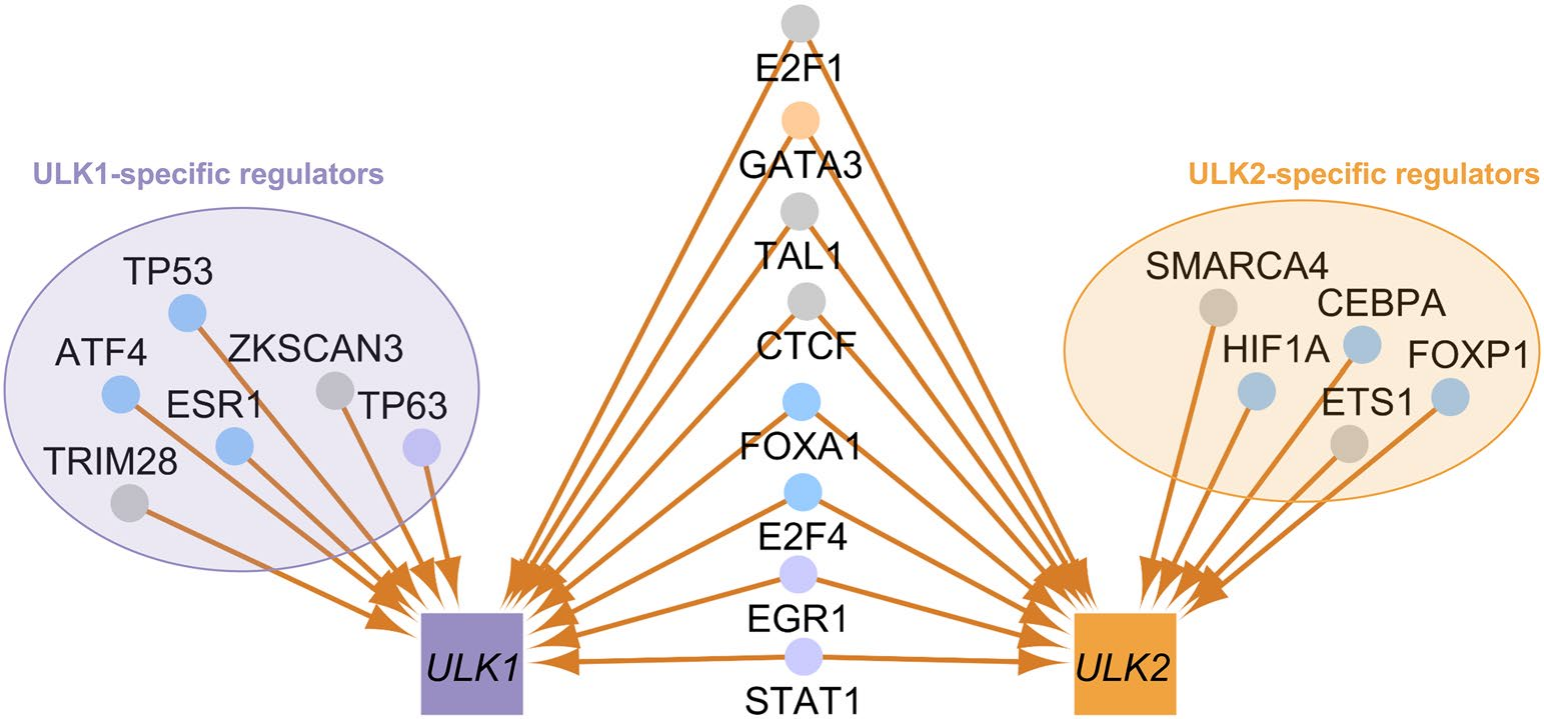
Experimentally validated transcriptional regulators of *ULK1* and *ULK2*. The nodes of the transcription factors are coloured based on the result of the transcription factor binding site (TFBS) analysis: Light purple: predicted TFBS supporting the experimental data was found on the *ULK1* promoter sequence; blue: predicted TFBS was found on the *ULK1* and *ULK2* promoter sequences; light orange: predicted TFBS was found on the *ULK2* promoter sequence; grey: no predicted TFBS was found.

We searched for experimentally verified transcription factors of *ULK1* and *ULK2* in databases and the literature by manual curation. Out of the 14 regulators of *ULK1* and 13 regulators of *ULK2*, six and five are specific, respectively. These transcription factors are likely to regulate the two *ULK* homologs differently. To extend the analysis, we predicted potential transcription factor binding sites (TFBSs) in the promoter regions of both *ULK* genes. We found that six transcription factors (TP53, ATF4, ESR1, HIF1A, CEBPA, FOXP1) that were experimentally identified as *ULK1-* or *ULK2*-specific, have actually binding sites along the other *ULK* gene as well, indicating potential new regulatory connections for experimental validation (Fig. 8). Those transcription factors whose TFBS was not found in the promoter region of *ULK1* or *ULK2* but previous experimental data showed the regulatory connection, are probably regulating the ULK gene’s expressions through more distant regulatory regions (e.g. in enhancer regions).

**Figure 9:**
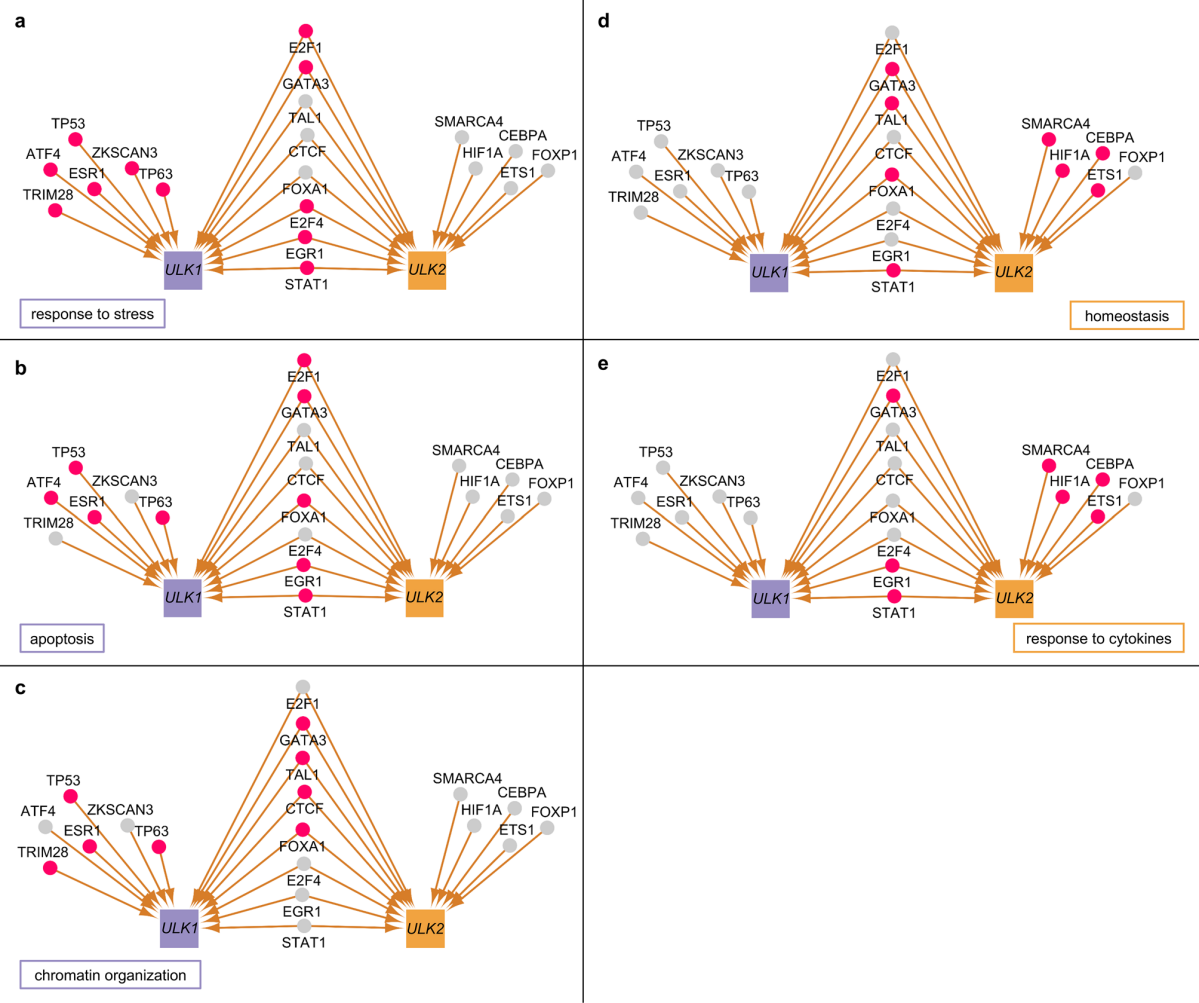
The transcriptional regulators of *ULK1* and *ULK2* and five biological processes with *ULK*-specific regulators. The specific biological processes are labelled on each network, transcription factors involved in the respective biological process are highlighted with pink nodes. (**a**–**c**) Shows processes predominantly specific to regulators of *ULK1*: (**a**) stress-related biological processes; (**b**) apoptosis; (**c**) chromatin organization; while (**d**, **e**) shows processes predominantly specific to regulators of *ULK2*: (**d**) homeostasis; (**e**) response to cytokines.

We also analysed the biological processes in which the transcription factors of *ULK1* and *ULK2* are involved. Based on a Gene Ontology Term Finder analysis^26^, we found that transcription factors of ULK1 are significant in stress response (Bonferroni corrected *P* value for the hypergeometric distribution < 0.0001), apoptosis (corrected *P* value < 0.0001) and chromatin organisation (corrected *P* value < 0.0001), while transcription factors of ULK2 seem to be important in homeostatic and immune system-related processes, including glucose homeostasis (corrected *P* value < 0.0001) and response to cytokines (corrected *P* value < 0.0001) (Fig. 9).

## Discussion

Here we report that despite being paralogs and the most similar in their secondary domain structure amongst the ULK homologs, the autophagy induction-related human ULK1 and ULK2 proteins are different in numerous aspects. The appearance of the *ULK1* and *ULK2* genes is the result of the duplication of the yeast *Atg1*. This duplication happened between the Deuterostomes and the Chordates, giving rise to two similar yet functionally different genes. With comprehensive bioinformatics approaches we show that ULK1 and ULK2 have specific protein interactors and transcriptional regulators, involved in distinct biological functions, supporting the specific roles of ULK1 and ULK2. Analysis of the interactors and regulators of ULK1 and ULK2 resulted in the identification of stress response, apoptosis, chromatin organization and intracellular transport as significantly shared among ULK1 interactors, whereas interactors of ULK2 are involved in nitrogen compound metabolic pathways, homeostasis and response to cytokines.

As reported in the literature, whole-genome duplication(s) happened throughout vertebrate evolution. The first such event happened at the base of the vertebrates, after the split of *Ciona* from vertebrates 500 million years ago, but prior to the split of fish from tetrapods^27–29^. In accordance with this, we found that the duplication of the *ULK1* ortholog also happened after the split of *Ciona* from vertebrates, so it was likely not a separate event but part of the whole-genome duplication.

By comparing the kinase domains of ULK1 and ULK2 (including sequence alignments and superimposition of 3D structures), we found non-aligning regions. This dissimilarity could contribute to the differences in the substrates of the two kinases which we indeed can see as ULK1 and ULK2 both have specific downstream interactors.

As ULK1 and ULK2 both had been identified as part of the autophagy induction complex, the two proteins are often mentioned interchangeably. However, as some specific studies have already shown, there are functional differences between them, for example they have opposing effects in lipid metabolism^10^. The functional dissimilarity can be a consequence of the different interacting partners and transcriptional regulators that we identified based on databases and manual curation.

Within the autophagy machinery network, ULK1 and ULK2 have 7 shared interactors, however five and two interactors are specific to ULK1 and ULK2, respectively, suggesting potential functional differences. As a specific example, we showed that these interactors were implicated in different types of selective autophagy: the interactors of ULK1 are more afftected in mitophagy, while WIPI2, the specific interactor of ULK2, is affected in xenophagy. In tissues where *ULK1* or *ULK2* is not expressed, or in conditions affecting one or the other paralog, these different types of selective autophagy could be impaired.

Regarding non-autophagy related protein–protein interactions, we found 14 common, 11 ULK1-specific and 21 ULK2-specific interactors. We assessed this interactor list with a domain-motif interaction analysis, and identified proteins amongst the experimentally validated ULK1- and ULK2-specific interactors which are in fact predicted to be capable of binding to both ULK1 and ULK2. We note that as we checked the source publication of each interaction we extracted from databases, we found that in many cases only ULK1 or ULK2 was investigated, making the specificity of an interactor to ULK1 or ULK2 questionable. Therefore, we provide a set of proteins that are predicted to bind to both ULK homologs but have not yet been shown in vitro to interact for future experimental validation studies. In our analysis we rely on experimentally verified interaction data, hence the uncertainty of the specificity becomes a limitation to our computational study as it could affect the functional gene ontology analysis. Some of our results are nonetheless supported by the literature, hence we believe our findings can unravel the so far hidden differences of ULK1 and ULK2.

We analyzed how *ULK1*, *ULK2* and the genes encoding their specific protein interactors are expressed in several tissues, to be able to put their interactions into the context of tissue specificity. Based on tissue expression levels of the interactors, ULK1 and ULK2 seem to have different specific interactor subgroups in different tissues. For example, *ULK1* has similar expression levels with *TMEM173*, *SMCR8*, *TRAF6* and *USP10* in the urinary bladder, while in pancreas it is more similar to *DYNLL1* and *PPP1CA*.

Beside protein–protein interactions we also collected transcriptional regulators of the two *ULK* homologs, and annotated transcription factor binding sites in one or both of the *ULK* homolog promoters. However, as the regulatory connections were mostly determined in high-throughput studies, in most cases we do not know if the specific transcription factors are actually specific to the respective *ULK* gene. The exceptions are ETS1 and p53. This is because Gao et al. analysed the effect of p53 on both *ULK* genes, and found that ectopic expression of p53 results in elevated level of *ULK1*, but not *ULK2*^30^. Interestingly, for p53, we found two transcription factor binding sites on the promoter of *ULK1*, but we also found one binding site along the promoter of *ULK2*. It is possible that because of the two transcription factor binding sites, p53 is more likely to bind to the promoter of *ULK1*, hence affecting its expression. Beside p53, other *ULK1-* or *ULK2*-specific transcription factors (ATF4, ESR1, CEBPA, HIF1A, FOXP1) have binding sites on the other *ULK* homolog as well, which makes them a potential pool for further experimental validation.

To investigate the non-autophagy related functional differences between ULK1 and ULK2, we analysed their protein interactors and transcriptional regulators for different biological processes and compared the results with the identified ULK1- and ULK2-specific protein motifs. ULK1 and ULK2 had already been found to be partially redundant, as for example double knockdown of *ULK1* and *ULK2* results in a severe reduction of glucose consumption in HCT116 cells, whereas *ULK1* and *ULK2* single knockdowns result in moderate reduction^31^. Nonetheless, *ULK1*, but not *ULK2* knockdown reduces the glucose consumption significantly^31^. This is consistent with our observations that ‘organic substance catabolic process’ and ‘carbohydrate metabolic process’ is a significant GO term shared between interactors of ULK1 but not ULK2. Interactors of ULK1 have also been found to have interactors sharing the function ‘Intracellular transport’. Among the affected interactors, SEC23A is known to be required for the translocation of insulin-induced glucose transporter SLC2A4/GLUT4 to the cell membrane in mouse fibroblasts^32^, which links ULK1 to glucose transport too. Another ‘Intracellular transport’-affected partner of ULK1, DYNLL1 regulates apoptotic activities of BCL2L11 by sequestering it to microtubules^33^.

In another study, Ro et al. showed that 3T3-L1 cells (a cell line model for adipocytes) do not require *ULK1* for adipogenesis, while knockdown of other autophagy genes, including *ULK2,* suppressed adipogenesis^10^. *ULK2* expression was upregulated in *ULK1* knockdown cells, and vice versa, but in *ULK2* knockdown cells upregulation of *ULK1* did not rescue the phenotype. In *ULK2* knockdown cells the expression of *PPARG* and *CEBPA* was decreased too, which also supports our results as CEBPA is one of the *ULK2*-specific transcription factors we highlighted.

We identified that protein interactors of ULK1 but not ULK2 were annotated with the process of stress response, which is in agreement with the results from Wang et al*.*, where they showed that stress granule proteins were enriched in the ULK1 interactome^34^. For creating an ULK1 interactome they used quantitative mass spectrometry and data from the STRING, BioPlex, InWeb, and BioGRID databases. Except for the BioGRID database, these are different databases to the ones used in this study, making their finding an independent evidence contributing to our results. Even though they did not check the interactome of ULK2, they performed experiments with both proteins and showed that ULK1 and ULK2 localized to stress granules. However, depending on the examined cell line, different levels of colocalization was observed between the TIA1 protein (stress granule marker) and a respective ULK homologue. While murine embryonic fibroblasts (MEFs) seem to display a better colocalization of stress granules and ULK1 (compared to the colocalization of TIA1 and ULK2), the mouse myoblast C2C12 cell line seems to show the opposite^34^. Nonetheless, as interacting partners of ULK1 share the GO term for cellular response to stress, our hypothesis is that ULK1 could have a bigger influence on stress response than ULK2. The results of the functional analysis are further supported by the presence of a phosphorylation site (DOC_Cyclin_RxL_1: Cyclin docking motif), specific to ULK1, on which cyclin/Cdk complexes involved in different biological processes, such as stress response can influence ULK1 (Table 1).

Importantly, our functional analysis suggests that ULK1 and not ULK2 has a specific role in apoptosis and programmed cell death as we found the apoptotic process being one of the significant GO terms that is shared among the transcription factors of ULK1. A crosstalk between apoptosis and autophagy is already well-described, but the difference between ULK1 and ULK2 in the process has not yet been defined^35^. We found ULK1-specific differences in relation to apoptosis on the protein interaction level: we identified an ULK1-specific motif (CLV_C14_Caspase3–7), which is annotated as a site for Caspase-3 and Caspase-7 cleavage. Kim et al. examined another autophagy-related protein, Beclin-1, and found that it is cleaved by caspases^36,37^. Strikingly, Beclin-1 contains the same protein motif we identified on ULK1, confirming that ULK1 could be indeed affected by apoptosis. Validation of the distinct effects of ULK1 and ULK2 on apoptosis could also shed light on further details of the fine regulation between apoptosis and autophagy. Supporting the importance of ULK1 in apoptosis, there is literature evidence that ULK1 acts as an anti-apoptotic protein however to our knowledge no information is available about ULK2^38,39^. The promoting or inhibiting relationship between ULK1 and apoptosis could also be more complex than it seems, if we consider the results of our protein motif analysis where we found that ULK1, but not ULK2, has a motif (LIG_BIR_III_2) responsible for binding to inhibitor of apoptosis proteins (IAPs). IAPs are known to negatively affect apoptosis^40^, and binding to IAPs can promote apoptosis by antagonizing their interactions with proapoptotic proteins such as caspase-9 and Smac^41^. We also found a motif on ULK2 (LIG_EF_ALG2_ABM_2) that was previously annotated as being capable of binding to ALG-2 protein in a Ca2 + -dependent manner. ALG-2 has been implicated in ER-stress-induced apoptosis, and shown to be pro-apoptotic^42,43^. This leads us to speculate that contrary to ULK1, ULK2 can in fact have a negative effect on apoptosis, however there is not yet an experimental evidence to support this hypothesis. To study the potentially complex effects of ULK proteins on apoptosis regulation, it would be crucial to study both ULK1 and ULK2 in the same experimental setup, and for example conducting an IAP and ALG-2 co-localisation assay with *ULK1/ULK2* single and double knockout cell lines. Moreover, following such cell line experiments, high throughput functional apoptosis assays could be conducted using available flow cytometry techniques for example developed by Thermo Fisher Scientific.

*ULK2*, opposed to *ULK1*, has previously been also shown to be essential for degradation of ubiquitinated proteins and homeostasis in skeletal muscle^16^. These results support our finding that transcription factors of *ULK2*, but not *ULK1,* are annotated to be significant in homeostasis. While our study is not specific for skeletal tissue, we show that *ULK2* definitely has the potential to have a greater impact on tissue homeostasis, which can be especially elevated in tissues with higher expression of *ULK2* than of *ULK1*.

Regarding further specific motifs, ULK2 harbours a TRAF6 binding site (LIG_TRAF6 motif), which is responsible for a response to the tumor necrosis factor receptor (TNFR) superfamily, and direct interaction with various TNF cytokine receptors. The presence of the TRAF6 binding site motif on ULK2 but not on ULK1 underlines the finding that *ULK2*-specific transcriptional regulators share the function for response to cytokines. This seems relevant in inflammatory diseases, like ulcerative colitis (UC), where the level of cytokines is increased. Interestingly, as we found in two microarray datasets (GSE6731^44^, GSE53306^44,45^), *ULK2* is downregulated in colon biopsies from inactive UC compared to healthy patients (The lists of differentially expressed genes from the GSE6731^44^ and GSE53306^45^ are included in Supplementary Table S1). It is crucial to highlight that while in the case of functions shared by ULK1 and ULK2, ULK homologs can have a compensatory effect when one of the homologs is missing, but compensation of specific functions is not possible. In the case of UC for example, ULK-specific functions can cause an insufficient reaction to cytokines^43^.

To be able to list all functions that are affected by the difference between ULK1 and ULK2, it would be key to experimentally compare the two proteins in various functional assays. Our systems level computational analysis can be a great source for further experimental testing. In Table 3, we listed some of the findings of our computational analysis, together with supporting experiments from the literature. In cases of certain functions, where only one of the ULK genes/proteins has been tested it would be essential to extend the experiment with the other ULK homolog. This would be especially useful for example in studying apoptosis, where ULK1 has been shown to acts as an anti-apoptotic protein however to our knowledge no experimental comparison is available with ULK2^38,39^.

**Table 3:** Suggested practical differences between ULK1 and ULK2.

Suggested functional difference	Literature evidence	Reference ID
‘Organic substance catabolic process’ and ‘carbohydrate metabolic process’ is a significant GO term shared between interactors of ULK1 but not ULK2	In HCT116 cells *ULK1*, but not *ULK2* knockdown reduces the glucose consumption significantly	Li et al*.*, 2016^31^
Interacting partners of ULK1 share the GO term for cellular response to stress; presence of a phosphorylation site (DOC_Cyclin_RxL_1: Cyclin docking motif), specific to ULK1, on which cyclin/Cdk complexes involved in different biological processes, such as stress response can influence ULK1	MEFs display a better colocalization of stress granules and ULK1 (compared to the colocalization of TIA1 and ULK2) In MEFs the contribution of Ulk2 to genotoxic stress-induced alternative autophagy appears to be small	Wang et al., 2019^34^ Torii et al., 2020^46^
The apoptotic process is one of the significant GO terms that is shared among the transcription factors of ULK1	ULK1 acts as an anti-apoptotic protein however to our knowledge no information is available about ULK2	Wang et al*.*, 2018^38^; Dower et al*.*, 2018^39^
Transcription factors of *ULK2*, but not *ULK1,* are annotated to be significant in homeostasis	*ULK2*, opposed to *ULK1*, has previously been also shown to be essential for degradation of ubiquitinated proteins and homeostasis in skeletal muscle	Fuqua et al*.*, 2019^16^
ULK2 harbours a TRAF6 binding site (LIG_TRAF6 motif), which is responsible for a response to the tumor necrosis factor receptor (TNFR) superfamily, and direct interaction with various TNF cytokine receptors. The presence of the TRAF6 binding site motif on ULK2 but not on ULK1 underlines the finding that ULK2-specific transcriptional regulators share the function for response to cytokines	In two microarray datasets, *ULK2* is downregulated in colon biopsies from inactive UC compared to healthy patients	GEO: GSE6731^44^, GSE53306^45^

We listed differences between ULK1 and ULK2 based on our computational study and how these are supported by the literature.

In conclusion, with our computational analysis, we have shown that two homologs in the autophagy induction complex, ULK1 and ULK2 likely have specific roles in certain biological processes controlled and mediated by ULK-specific upstream and downstream protein interactors, and transcriptional regulators. In addition, interactors of ULK1 and ULK2 within the autophagy core machinery are involved in different types of selective autophagy, which can mean different outcome in term of selective autophagy when one of the ULK homologs is missing or affected for example in disease. In order to understand the practical consequence of the presented key differences between ULK1 and ULK2 on autophagy, experimental studies for disease conditions such as UC should consider analysing these two genes separately. Further analysis on this could help us understand more details about how autophagy is dysregulated in diseased conditions.

## Materials and methods

For the comparison of domain structures of the ULK homologs, we used the Pfam website (https://pfam.xfam.org/)^47^ and for comparison of 3D secondary structure of the ULK1 and ULK2 kinase domain we used the UCSF Chimera software (https://rbvi.ucsf.edu/chimera)^48^. For the annotation of the ULK1 gene duplication event, the dendrogram was adapted from the OMA orthology browser (https://omabrowser.org/)^49^: the branches were collapsed into representative groups and information about the number of homologs in the respective taxa was added. DNA and protein sequences were retrieved from Uniprot (https://uniprot.org/)^50^ and their protein alignments were carried out with MUSCLE (https://ebi.ac.uk/Tools/msa/muscle/)^51^ with default parameters and ClustalW output format. The protein motifs were downloaded from the Eukaryotic Linear Motif (ELM) resource (https://elm.eu.org/)^52^. The motifs on ULK1 and ULK2 went through a globular domain filtering, structural filtering and context filtering, so we analysed only the accessible motifs that are either outside of globular protein domains or they have an acceptable structural score. Tissue expression data was downloaded from The Human Protein Atlas (https://proteinatlas.org/about/download)^53^ as consensus RNA expression. The heatmaps were generated with the heatmap.2 function, with default clustering settings and column-scaling which was normalized to both ULK1- and ULK2-specific heatmaps. The promoter sequences of the human genes *ULK1* and *ULK2* were retrieved using the "retrieve sequence" function of RSAT (https://rsat.eu/)^54^. For the genome assembly of these genes we used the Genome Reference Consortium Human Build 38 patch release 13 (GRCh38.p13)^55,56^. The genome annotation was obtained with the “Full genome build” method (version GENCODE 19), and it contains protein-coding and non-coding genes, splice variants, cDNA, protein sequences and non-coding RNAs. The transcription factor binding site prediction was carried out using the RSAT "matrix-scan" tool and the JASPAR 2018 non-redundant matrices containing the binding profiles of human transcription factors (TFs) (https://jaspar2018.genereg.net/)^57^. Predicted sites with a *P* value of < 0.0001 were considered to be significant.

Interaction data was downloaded from databases on the 10th of July, 2019. We used various databases to collect experimentally verified, directed protein–protein (PPI) and transcription factor-target gene interactions for human: the Autophagy Regulatory Network, that involves manually curated interactions between autophagy proteins, their post-translational regulators and between signalling components obtained from manual curation, 19 external databases and 4 prediction methods (ARN, https://autophagyregulation.org/)^23^; OmniPath, containing 20 resources that provide causal interactions (12 about activity flow and 8 about enzyme–substrate interactions), 8 that deliver undirected interactions from both literature curation and high-throughput screens, and 6 that capture biochemical reactions (https://omnipathdb.org/)^58^; TRRUST, a manually curated database of human and mouse transcriptional regulatory networks derived from 11,237 Pubmed articles, which describe small-scale experimental studies of transcriptional regulations (version 2, https://grnpedia.org/trrust/)^59^; ORegAnno, that involves regulatory regions, TFBSs and regulatory variants (polymorphisms and haplotypes) from 19 species from external datasets and extensive manual curation, of ChIP-seq, DNase-seq, FAIRE-seq and other experiments (version 3.0, https://oreganno.org/)^60^ and HTRIdb, containing unique experimentally verified transcriptional regulation interactions detected by small and mid-scale techniques (chromatin immunoprecipitation, concatenate chromatin immunoprecipitation, CpG chromatin immunoprecipitation, DNA affinity chromatography, DNA affinity precipitation assay, DNase I footprinting, electrophoretic mobility shift assay, southwestern blotting, streptavidin chromatin immunoprecipitation, surface plasmon resonance and yeast one-hybrid assay) and by chromatin immunoprecipitation coupled with microarray (ChIP-chip) or chromatin immunoprecipitation coupled with deep sequencing (ChIP-seq)^61^. Additionally, we used PPI predictions based on the Pfam (https://pfam.xfam.org/)^47^ and ELM (https://elm.eu.org/)^52^ databases. The ARN contains PPI and TF-TG interactions specifically related to autophagy, whereas the other databases are more general, so we filtered the data to obtain a subset of interactions containing ULK1 and ULK2-related connections. In the final PPI dataset we included experimentally verified interactions only when they were directed, and interactions where information on the direction was not available from the experimental data but we obtained the direction by our domain-motif interaction prediction.

To obtain the significantly shared biological processes of interactors of ULK1 and ULK2, we used the Generic Gene Ontology Term Finder (GOTermFinder, https://go.princeton.edu/cgi-bin/GOTermFinder)^26^, and we filtered GO terms by being specifically shared among interactors of ULK1 or ULK2. As the GOTermFinder gives significant GO terms as a result, the corrected *P* value was below 0.01 for all GO terms. Microarray datasets containing biopsy samples from inactive UC and healthy patients (GSE6731^44^, GSE53306^45^) were analysed with the GEO2R function of the Gene Expression Omnibus (GEO, https://ncbi.nlm.nih.gov/geo/) with default settings. For network visualisation of the ULK1 and ULK2-related interactions we used the Cytoscape program (version 3.7.0, https://cytoscape.org/).

## Supplementary information

Supplementary Information

Supplementary Table S1

Supplementary Table S2

Supplementary Table S3

## Data Availability

The datasets generated and analysed during the current study are present in the Supplementary materials.
