# Supplementary material for: ULK1 and ULK2 are less redundant than previously thought: computational analysis uncovers distinct regulation and functions of these autophagy induction proteins
Source: Sci Rep. 2020 Jul 2;10:10940. doi: 10.1038/s41598-020-67780-2 (PMC7331686; doi:10.1038/s41598-020-67780-2)
Supplement: Supplementary file 1 — Supplementary Information [file 41598_2020_67780_MOESM1_ESM.docx]

**ULK1 and ULK2 are less redundant than previously thought: Computational analysis uncovers distinct regulation and functions of these autophagy induction proteins**

Amanda Demeter^1,2^, Mari Carmen Romero-Mulero^1,3^, Luca Csabai^1,4^, Márton Ölbei^1,2^, Padhmanand Sudhakar^1,2^, Wilfried Haerty^1^, Tamás Korcsmáros^1,2,*^

^1^Earlham Institute, Norwich Research Park, Norwich, NR4 7UZ, UK

^2^Quadram Institute Bioscience, Norwich Research Park, Norwich, NR4 7UQ, UK

^3^Faculty of Biology, University of Seville, Seville, 41012, Spain

^4^Eötvös Loránd University, Budapest, 1117, Hungary

^*^Corresponding author details:

Dr Tamás Korcsmáros

ORCID id: <https://orcid.org/0000-0003-1717-996X>

Email: [Tamas.Korcsmaros@earlham.ac.uk](mailto:Tamas.Korcsmaros@earlham.ac.uk)

Phone: 0044-1603450961

Fax: 0044-1603450021

Address: Earlham Institute, Norwich Research Park, Norwich, NR4 7UZ, UK

**Supplementary information**


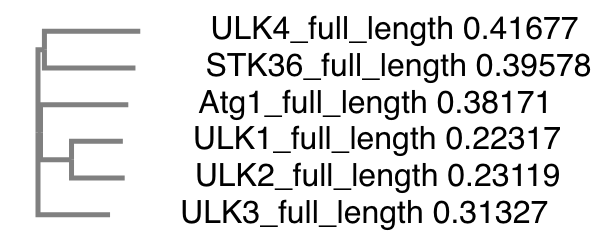


**Supplementary Figure S1: Phylogenetic tree of full the yeast Atg1 protein and its human homologs (complete protein).** Based on multiple sequence alignment of the whole length of the proteins, ULK1 and ULK2 are the most similar to each other.

**Supplementary Table S1: Differentially expressed genes from comparing microarray datasets containing biopsy samples from inactive UC and healthy patients (Datasets GSE6731 and GSE53306 from the Gene Expression Omnibus).** Log fold change values were calculated using the built-in GEO2R function of the GEO website. The table contains the calculated median of all values to the same gene (gene symbol), if the adjusted P value was above 0.1. The table contains only genes with abs(med_logFC) >= 0.585 and the highest adjusted P value was kept.

**Supplementary Table S2: Information about experimentally validated PPI and TFTG interactions. Details** on each of the interactions shown in Figures 4, 5 and 8. Included details are: Source name (Column A): Gene symbol of the source interaction partner, Target name (Column B): Gene symbol of the target interaction partner, Source Uniprot ID (Column C), Target Uniprot ID (Column D), Interaction type (Column E): PPI directed/undirected/directed by prediction or Transcriptional regulation, Detection method (Column F), Cell type/tissue (Column G), Pubmed ID (Column H): reference ID of the article describing the experiment, Source database (Column I): original database including the interaction, Downloaded from (Column J): the database where the interaction was downloaded from or manual curation, ULK investigated (Column K): information of the specificity of the interaction for example if they tested both ULK1 and ULK2 or not.

**Supplementary Table S3: Expression values of ULK1 and ULK2 in different tissues.** Consensus normalized RNA expression ("NX") values were downloaded from Human Protein Atlas.
